# Sugar intake trajectories in adolescents: Evaluating behavioral change with group-based trajectory modeling

**DOI:** 10.1371/journal.pone.0333389

**Published:** 2025-09-30

**Authors:** Jisu Lee, Hyeonkyeong Lee, Sun Young Shim, Chang Gi Park, Hyeyeon Lee

**Affiliations:** 1 Mo-Im Kim Nursing Research Institute, College of Nursing, Yonsei University, Seoul, South Korea; 2 Institute for Innovation in Digital Healthcare, Yonsei University, Seoul, South Korea; 3 Department of Population Health Nursing Science, College of Nursing, University of Illinois Chicago, Chicago, Illinois, United States of America; 4 College of Nursing, Kosin University, Busan, South Korea; Lusofona University of Humanities and Technologies: Universidade Lusofona de Humanidades e Tecnologias, PORTUGAL

## Abstract

**Background:**

Sugar intake through sugar-sweetened beverages (SSBs) remains a major public health concern among adolescents. Tailored dietary interventions have garnered interest for promoting sustainable behavior change, yet traditional pre-post designs often overlook the temporal complexity of individual adaptation. This study applied a trajectory-based approach to assess how adolescents’ sugar intake trajectories evolved in response to an intake-based tailored intervention.

**Methods:**

A secondary analysis was conducted on data from a 14-day chatbot intervention (CRIS, KCT0008114) aimed at reducing sugar intake among adolescents. Group-based trajectory modeling (GBTM) was used to identify distinct intake trajectories. A linear mixed-effects model examined the impact of tailored intervention type, time, and trajectory group membership on sugar consumption, including their interactions. Subgroup analysis compared intervention responses between native Korean and racial and ethnic adolescents.

**Results:**

Three trajectory groups were identified: reduction (38%), maintenance (57%), and no-intake (5%). Adolescents with higher baseline intake exhibited rapid declines in consumption, whereas those with lower intake showed gradual reductions. By the second week, reduction and maintenance groups converged. A significant three-way interaction among intervention type, time, and trajectory group was observed, indicating heterogeneous responses. No group exhibited increased consumption, suggesting sustained effects. Racial and ethnic adolescents demonstrated greater responsiveness to the tailored intervention.

**Conclusion:**

Intake-based tailored interventions effectively accommodate individual variability in dietary behavior change, with particularly pronounced benefits among participants with higher baseline intake. These findings underscore the importance of adaptive intervention strategies and the need to consider individual and structural factors when designing public health interventions targeting adolescent diet.

## Introduction

Sugar-sweetened beverages (SSBs) consumption has significantly increased [1], contributing to serious health risks, such as metabolic syndrome and cancer [2], dental erosion [3], mental health problems [4,5] and substance use [6]. Notably, the high accessibility and popularity of carbonated and energy drinks have contributed to excessive sugar intake among adolescents [7], making it a pressing global public health concern. Particularly, racial and ethnic adolescents face higher vulnerability due to limited access to health information or services [8], placing them at greater risk of adverse health outcomes. Despite concerted efforts, traditional one-way mass-media campaign has not produced durable reductions in SSB consumption [9], and interactive SMS-based chatbot intervention also likewise yield only short-term gains that diminish during long-term follow-up [10]. These findings highlight the need for more immediate and individualized interventions to regulate SSB consumption behaviors in adolescents’ daily lives.

Effective dietary interventions require the provision of just-in-time, personalized, and actionable feedback [11], which has been shown to be essential for promoting sustained behavioral change. In this context, chatbots have emerged as promising tools that address the limitations of traditional interventions by enabling real-time monitoring and feedback provision [12]. Previous studies have demonstrated chatbots’ effectiveness in facilitating dietary improvements and weight management [13], highlighting their potential as tailored intervention strategies to enhance the efficacy of digital health interventions. To optimize these benefits, adaptive intervention strategies [14], have been proposed to dynamically adjust feedback and intervention intensity based on individual responses.

Existing studies have predominantly employed pre-post experimental designs to assess changes in SSB consumption, averaging outcomes across groups rather than capturing individuals’ responses, thereby limiting the ability to analyze individual effects [15,16]. Given the variability in individual responses to dietary factors, a more comprehensive approach is required to evaluate longitudinal patterns of consumption [17]. To address this limitation, dietary trajectory analysis has been proposed as a methodological approach to better assess individual variations in dietary behavior over time [18]. This analytical approach enables researchers to track changes in SSB consumption in response to real-time interventions, thereby providing a more precise understanding of intervention effectiveness. Moreover, dietary trajectory analysis facilitates the development of personalized intervention strategies, allowing for the refinement of digital health interventions to achieve more sustainable behavioral outcomes.

Group-based trajectory modeling (GBTM) is a trajectory analysis method used to determine the groups of heterogeneous change trajectories over time and has been widely applied in various fields, including dietary pattern analysis, psychology, and pharmacology [19]. Given that sugar consumption do not follow a linear trajectory [20], GBTM serves as a powerful tool for analyzing how individuals respond differently to personalized interventions over time [18]. Furthermore, identifying distinct sugar intake trajectory group allows for the evaluation of whether intervention effects are sustained, gradually diminished, or lead to specific behavioral adaptations. Addressing this research gap will contribute to developing more sustainable and effective intervention strategies aimed at reducing sugar consumption.

This study aimed to examine how adolescents’ sugar intake behaviors evolved over time in response to an intake-based tailored sugar reduction intervention. By applying GBTM, we identified distinct sugar intake trajectories and explored how tailored intervention influenced behavioral adaptation within each trajectory group. In light of evidence that racial and ethnic adolescents derive less benefit from generic interventions [21], we also conducted an exploratory analysis of potential cultural disparities in intervention receptivity by comparing racial and ethnic adolescents with the overall cohort.

## Methods

### Study design, data sources, and participants

This study conducted a secondary analysis of data originally collected during a 14-day usability study of the chatbot intervention, “R-Ma Bot”, which aimed to reduce the consumption of carbonated and/or energy drinks [22]. Access to the anonymized dataset commenced on 14 February 2025 upon receiving IRB approval, and data analysis was completed by 31 March 2025. The original data were collected from a single-group sample of 42 adolescents residing in South Korea: 21 Korean native adolescents, whose parents were both born in Korea and hold Korean nationality, and 21 racial and ethnic adolescents, defined as those from families in which at least one foreign-born parent. Participants were recruited by convenience sampling at two middle schools in Seoul or Incheon: flyers were posted with school permission, researchers held brief on-site information sessions, written parent–student consent was obtained, and enrollment closed once each subgroup (n = 21) reached the target calculated a priori for statistical power. In the original study, adolescents were eligible to participate if they were enrolled in middle school and were proficient in reading, writing, and speaking Korean. Adolescents with medical conditions requiring dietary therapy or medication were excluded from the original study.

### Intervention: R-Ma Bot

R-Ma Bot is a chatbot designed to support the reduction of sugar, sodium, and caffeine intake from carbonated and/or energy drinks through the application of behavior change techniques (BCTs). The intervention lasted for 14 days, during which the chatbot provided personalized feedback by comparing daily intake levels of each ingredient, guiding participants in making behavioral adjustments. Further details of the R-Ma Bot intervention are described in the original study [22].

The intervention delivered two types of intake-based tailored feedback: reward-based and education-based feedback, tailored according to the participant’s intake patterns for each nutrient. Participants who reduced or maintained their intake of sugar, sodium, or caffeine received reward-based feedback, while those who increased their intake of any of the three components were provided with education-based feedback.

For reward-based feedback, points were assigned separately for each ingredient (sugar, sodium, and caffeine) based on the extent of intake reduction compared to the previous day. A *strong reward of 200 points* was awarded when the participants reduced their intake of a specific ingredient. *A moderate reward of 100 points* was granted when a participant completely abstained from consuming that ingredient for two consecutive days. *A weak reward of 50 points* was given when the intake of a specific ingredient remained stable (not an increase or decrease) compared to the previous day.

Conversely, when intake increased, education-based feedback was provided instead of rewards, delivered through infographic slides and progressing through three distinct phases if the increase persisted. *Basic education* applied the ‘Information about health consequences’ strategy to raise awareness of the adverse effects of excessive intake. If intake continued to increase the next day, *Advanced education* introduced the ‘Information about others’ approval’ strategy, which provided information on recommended intake levels and the content of these ingredients in various beverages. If further intake increases were observed, *Specific education* implemented the ‘Behavior substitution’ strategy, suggesting alternative beverage options with lower sugar, sodium, or caffeine content to encourage healthier choices.

### Measurements

#### Sugar intake.

In this study, only the sugar intake over the 14-day period was extracted as a dependent variable and analyzed. Daily sugar intake was calculated based on the types and quantities of carbonated and energy drinks consumed, which were recorded each night. The sugar content per serving of each beverage was obtained from the beverage database provided by the Ministry of Food and Drug Safety [23].

#### Intervention type.

The intervention types were categorized into six feedback: strong reward, moderate reward, weak reward, basic education, advanced education, and specific education. To assess the effect of these interventions, the variable was extracted as a lagged variable, utilizing data from the 13 days, excluding the final day (day 14). If no sugar intake data were entered, the intervention was not delivered, and the data was marked as missing.

However, during the 14-day intervention period, no participants received the ‘specific education’ feedback. Therefore, this type was excluded from the final analysis due to the absence of observed cases.

#### General characteristics.

General characteristics included gender, grade, age, academic status, socioeconomic status, parental foreign status, height, and weight. Grade referred to the participant’s current school grade (7th–9th). Academic status was self-rated on a 5-point scale (1 = very low to 5 = very high) and dichotomized at the midpoint (scores ≥ 3 = good, < 3 = poor). Subjective socioeconomic status was recorded on a 10-point ladder (1 = lowest, 10 = highest) [24] and dichotomized as lower (< 5) versus higher (≥ 5) status. Parental foreign status was derived from the reported country of birth of each parent and coded as two categories: one foreign parent versus both parents’ foreign nationals. The body mass index (BMI) was calculated based on participants’ height and weight. These characteristics were collected through a pretest survey.

### Statistical analysis

The general characteristics of all participants were calculated as N (%), mean ± SD. Differences in continuous variables were analyzed using a t-test, while differences in categorical variables were analyzed using the chi-square test or Fisher-Freeman-Halton exact test.

Sugar intake trajectories were modeled for 42 participants over a 14-day period based on self-reported sugar intake data. GBTM approach was employed to identify subgroups with similar patterns of sugar intake. The number of trajectory groups progressively increased, starting with two, until the model failed to converge. The optimal number was determined based on model fit [19]. Model fit was evaluated using two criteria, both generated automatically by the ‘gbmt’ function in R and extracted from the model output: (1) the Bayesian Information Criterion (BIC) with lower values indicating a better fit and (2) the average posterior probability of assignment (APPA)—the mean posterior probability for individuals assigned to a given group—of ≥ 70%. Each group was also required to include at least 5% of the participants.

A linear mixed-effects model was used to evaluate the intervention effect over time, with a random intercept accounting for individual-level variation in baseline sugar intake. Trajectory group, gender, age, socioeconomic status, academic status, and BMI were included as person-level (between-subject) fixed effects, whereas time and intervention type—calculated each day from the previous- and current-day intake (lagged)—were treated as day-level (within-subject, time-varying) fixed effects. To examine whether the intervention effects differed over time and across trajectory groups, interaction terms were specified, including two-way interactions (intervention type*time, intervention type*group), and a three-way interaction (intervention type*time*group). The intervention effect was quantified using the parameter estimate with the respective 95% Wald confidence intervals. A subgroup analysis was conducted to explore intervention differences between Korean native and racial and ethnic adolescents by repeating the main analysis separately within each group.

GBTM was conducted using the ‘gbmt’ package in R version 4.3.2, while other analyses were implemented in SAS version 9.4 (SAS Institute Inc., Cary, NC, USA). With about 5% missingness limited to the outcome, models were fitted by likelihood‑based methods under the Missing at Random (MAR) assumption, using all available data and excluding only outcome‑missing time points.

### Ethics approval

Exempted review approval for this secondary analysis (IRB No. 4-2024-1600) was granted by the institutional review board (IRB) that had approved the original study. During the original study, written informed consent was obtained from all adolescent participants and their parents. This consent explicitly included permission for future reuse of anonymized data. For the current study, all data were fully de-identified prior to access, and the research team had no access to personally identifiable information at any stage of the analysis. The reuse of this data adhered to ethical guidelines for secondary data analysis.

## Results

### General characteristics of study participants

Table 1 describes the general characteristics of the study participants (N = 42). Over two-thirds of the participants were girls (n = 29, 69.0%), with a mean age of 16.0 ± 0.7 years. Most participants (n = 30, 71.4%) reported good academic performance (average or above) and the same portion perceived their socioeconomic status as relatively low (below middle). Regarding sugar intake, the overall mean intake was 10.49 ± 15.09 g/day, with higher intake in Week 1 (13.11 ± 20.13 g/day) compared to Week 2 (7.86 ± 12.78 g/day). The most frequently consumed beverage was Coke, accounting for the largest proportion of total sugar intake.

**Table 1:** General characteristics of participants (N = 42).

Variables	Category	Total		Native Korean		Racial and ethnic		*p*
		(N = 42)		(n = 21)		(n = 21)		
		n (%) or mean±SD						
Gender	Boy	13	31.0	4	19.1	9	42.9	.182
	Girl	29	69.0	17	81.0	12	57.1	
Grade	7th	12	28.6	6	28.6	6	28.6	.024
	8th	21	50.0	14	66.7	7	33.3	
	9th	9	21.4	1	4.8	8	38.1	
Age (year)		16.0	0.7	15.8	0.6	16.1	0.8	.209
Academic status	Good	30	71.4	14	66.7	16	76.2	.733
	Poor	12	28.6	7	33.3	5	23.8	
Socioeconomic status	<Middle	30	71.4	17	81.0	13	61.9	.306
	≥Middle	12	28.6	4	19.1	8	38.1	
Parental foreign status	One foreign-born parent	6	28.6	–	–	6	28.6	–
	Two foreign-born parents	15	71.4	–	–	15	71.4	
Height (cm)		164.4	9.6	164.6	8.5	164.1	10.8	.863
Weight (kg)		55.3	12.2	54.8	12.6	55.8	12.1	.804
Body mass index (kg/m^2^)		20.3	3.0	20.0	3.3	20.5	2.8	.611
Sugar intake	Overall	10.49	15.09	8.77	14.32	12.20	15.99	.467
	Week 1	13.11	20.13	9.38	18.06	16.83	21.80	.235
	Week 2	7.86	12.78	8.15	12.66	7.57	13.21	.886

Note. *p*-values represent the results of chi-square tests and Fisher-Freeman-Halton exact test using Monte Carlo simulation (10,000 replicates) for categorical variables and independent t-tests for continuous variables, comparing Korean native and racial and ethnic adolescents.

The comparison of general characteristics between Korean native and racial and ethnic adolescents revealed that both groups were largely homogeneous, except for grade distribution (*p* = .024). Among Korean native adolescents, only one participant was in the third year, with the majority concentrated in the second year, whereas racial and ethnic adolescents were more evenly distributed across all grades. Additionally, no significant differences were observed in sugar intake between the two groups.

### Sugar intake trajectories in response to the intervention

**Fig 1:**
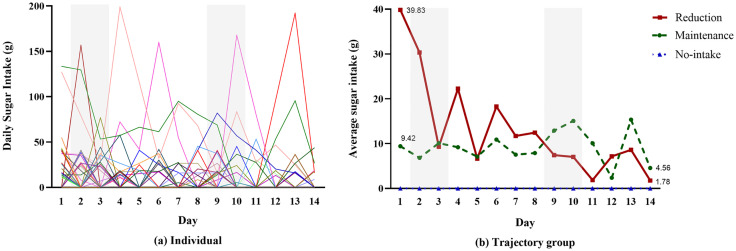
Sugar intake trajectories in response to the intervention. Note. Shaded areas represent weekend days (Saturday and Sunday) during the 14-day monitoring period.

GBTM identified three distinct sugar intake trajectories among the 42 participants over the 14-day intervention period (Fig 1b): reduction (n = 16, 38.1%), maintenance (n = 24, 57.1%), and no-intake (n = 2, 4.8%). There was no significant difference in the distribution of participants across sugar trajectory groups between the native Korean group and the racial and ethnic group (*p* = .871). These trajectories highlight individual variability in response to the intervention, suggesting that behavior change patterns are not uniform but instead follow distinct adaptation pathways. Notably, substantial daily fluctuations and inter-individual differences in sugar intake were observed, as illustrated by individual trajectories (Fig 1a).

The reduction group exhibited a sharp decline in sugar intake, with an average daily intake of 13.38 ± 29.88 g/day, dropping significantly from 39.83 ± 39.99 g/day at baseline to 1.78 ± 6.89 g/day by the end of the intervention. In contrast, the maintenance group showed a gradual decrease, maintaining an overall average intake of 9.23 ± 22.09 g/day, with sugar consumption declining from 9.42 ± 14.56 g/day on Day 1 to 4.56 ± 10.51 g/day on Day 14. The no-intake group remained completely abstinent from sugar intake throughout the study period, following a consistently non-consumptive trajectory.

The robustness of these trajectory classifications was confirmed by average posterior probabilities ≥0.70 (reduction: 0.730, maintenance: 0.859, no-intake: 1.000), indicating a strong model fit (see S1 Table). These findings emphasize that intervention responses varied significantly across individuals, supporting the need for trajectory-based analyses to capture the complexities of dietary behavior change.

### Effect of the tailored intervention on sugar intake trajectories

A linear mixed-effect model was applied to examine how tailored interventions influenced sugar intake behaviors over time, incorporating trajectory groups included as moderators (Table 2).

**Table 2:** Estimated effect of the intervention based on linear mixed effect model.

Variables	Total (n = 42)			Korean Native (n = 21)			Racial and ethnic (n = 21)		
	β	SE	*p*	β	SE	*p*	β	SE	*p*
Intercept	32.35	73.69	.663	−187.61	111.62	.117	214.98	118.55	.093
Intervention type									
Strong reward	13.06	17.43	.454	−13.32	19.01	.484	31.29	47.93	.515
Moderate reward	9.63	17.67	.586	−17.01	18.82	.367	30.83	48.39	.525
Minimal reward	38.48	183.37	.834	120.11	163.57	.464	−4.32	52.08	.934
Basic education	−2.43	17.22	.888	−23.48	18.88	.215	10.41	47.64	.827
Sugar intake trajectories									
Reduction	21.26	11.25	.059	5.23	12.20	.669	32.05	19.60	.104
No-Intake	−5.18	18.38	.778	−3.17	21.39	.882	−9.71	31.32	.757
Gender									
Girl	1.24	7.43	.868	−7.08	11.21	.528	10.13	10.71	.346
Age (year)	−2.80	4.52	.536	12.90	7.08	.070	−14.73	7.10	.039
Socioeconomic status									
Low	−1.00	7.04	.887	2.95	10.33	.775	−8.44	10.82	.436
Academic status									
Low	−5.06	6.99	.470	−10.10	9.80	.304	−19.16	13.36	.153
Body mass index (kg/m^2^)	1.40	1.08	.198	0.15	1.30	.906	2.27	1.83	.218
Intervention type*time			.001			.022			.005
Intervention type*group			.775			.953			.628
Intervention type*time*group			.001			.187			.014

Note. Reference groups: Intervention type (advanced education), Sugar intake trajectories (maintenance), Gender (boy), Socioeconomic status (≥moderate), Academic status (≥moderate). *Intervention type* refers to six types of tailored-feedback via the R-Ma Bot during the 14-day period. *Group* refers to the sugar intake trajectory group (maintenance, reduction, or no-intake).

Among all adolescents (N = 42), none of the specific intervention types (i.e., strong reward, moderate reward, minimal reward, or advanced education) demonstrated a statistically significant effect on sugar intake compared to the advanced education reference group (all *p* > .050). Similarly, the main effects of sugar intake trajectory groups (reduction and no-intake) were not statistically significant (*p* > .050). In contrast, the intervention type exhibited a significant interaction with time (*p* = .001), indicating a cumulative change in sugar intake across the 14-day period. Moreover, the three-way interaction among intervention type, time, and sugar intake trajectory group was also significant (*p* = .001), suggesting that the effectiveness of the intervention differed according to adolescents’ sugar intake trajectories during the intervention period.

Subgroup analysis indicated cultural differences in intervention receptivity. Among racial and ethnic minority adolescents (n = 21), sugar intake showed significant changes over time (intervention type*time, *p* = .005), and these changes significantly differed by sugar intake trajectory group (intervention type*time*group, *p* = .014). Additionally, older age was associated with a greater reduction in sugar intake (β = −14.73, *p* = .039). When parental foreign status (one vs. both foreign parents) was added as a covariate, the results remained consistent with those observed in this subgroup (see S2 Table).

In contrast, among Korean native adolescents, although a time-dependent effect of the intervention type was observed (*p* = .022), there was no evidence that sugar intake trajectories moderated this effect (intervention type*time*group, **p* *= .187), suggesting more uniform responses to the intervention across trajectory groups.

## Discussion

This study examined how adolescents’ sugar intake behaviors changed in response to an intake-based tailored sugar reduction intervention. By applying GBTM, we identified three distinct sugar intake trajectories: reduction, maintenance, and no-intake groups. Unlike traditional pre-post analyses that provide a static assessment of intervention effects [25], the trajectory-based approach captured the dynamic and individualized nature of dietary behavior change, offering deeper insights into how dietary habits adapt over time. The significant three-way interaction among intervention type, time, and trajectory group highlights that individual with different intake trajectories responded uniquely to the intervention, reinforcing that behavioral adaptation is not uniform; rather, it depends on pre-existing consumption status and individual response variability.

The reduction group exhibited an immediate decline in sugar intake, whereas the maintenance group showed a gradual adaptation pattern. On the first day, participants in the reduction group consumed approximately 40 g of sugar per day, significantly higher than the 9 g observed in the maintenance group. This suggests that individuals with higher baseline intake may be more responsive to tailored intervention providing rewards and education, possibly due to heightened awareness of excessive consumption or stronger motivation to change. As explained in common health behavior theories such as Transtheoretical model [26] and Protection motivation theory [27], individuals are more likely to change their behavior when they perceive health risks and believe that their actions can mitigate them. Therefore, providing health information that raises awareness of the risks associated with SSB consumption is crucial. Other research suggests that populations with limited access to health information face greater challenges in acquiring and applying health-related knowledge [28], making tailored interventions particularly beneficial. The findings of this study further support the role of tailored interventions in addressing health information disparities.

Despite differences in temporal patterns of response, trajectory groups did not significantly moderate overall intervention effectiveness. Instead, the three-way interaction indicates that intervention effectiveness varied within trajectory groups over time rather than being predetermined by baseline intake levels. Although GBTM effectively classified distinct consumption patterns, baseline trajectory group membership alone was not a decisive factor in determining intervention responsiveness. Consistent with earlier trajectory research—showing that sugar intake can worsen through cumulative exposure [29]—this finding underscores the need for targeted strategies that explicitly leverage trajectory information. Consequently, real-time adaptive interventions that dynamically adjust feedback to evolving consumption behaviors [14] are likely to outperform one-directional educational formats [10]. Moreover, because GBTM captures the dynamic nature of behavior over time, the two-week observation period in this study limits the ability to fully characterize these patterns. Future long-term intervention studies are warranted to clarify the trajectories of behavioral patterns resulting from individualized interventions.

Although the effects of each intervention type were not statistically significant, the intake-based tailored intervention demonstrated a significant overall effect in reducing sugar intake over time, reflecting a cumulative and time-dependent impact. This suggests that dynamic intervention strategies may be more effective than static, one-time educational approaches. Such findings align with the reinforcement learning framework [30], which posits that behavioral change is not driven by singular decisions but shaped by the accumulation of repeated reinforcement experiences over time. In the present study, tailored intervention may have functioned as a regulatory mechanism responsive to participants’ behavioral trajectories, potentially preventing relapses in the reduction group and supporting stable intake in the maintenance group. However, complete cessation of SSBs consumption remained challenging, indicating the need for more intensive or sustained intervention efforts. In fact, the participants’ sugar intake from SSBs may be overlooked simply because it remained below the American Heart Association (AHA) recommendation of 25 g per day [7]. Considering the substantial contribution of SSB to overall sugar intake [31], their associated health risks [32] and the habitual consumption patterns of SSB [33], adolescents’ intake is likely to exert long-term effects across the lifespan. Thus, sustained enforcement in future interventions to change the SSB intake is essential to mitigate these effects. As the educational environment becomes increasingly digitalized, integrating digital self-recording applications into school health services will enable students to collect their daily beverage intake. This data can be sent to chatbots that provide real-time, personalized feedback, and educators can tailor nutrition education based on observed intake profiles.

Another notable finding of this study was the differential impact of the intervention between Korean native and racial and ethnic adolescents. While both groups exhibited sugar intake reduction over time, the intervention alone significantly impacted only racial and ethnic adolescents. These disparities may be attributed to differences in health literacy and access to nutritional information. Prior studies indicate that marriage-migrant women in Korea tend to have lower health literacy [34], with limited access to health information, language barriers, and cultural differences, all of which may hinder their ability to guide their children’s dietary habits [35]. As a result, racial and ethnic adolescents may have received less structured dietary education at home or school, making them more responsive to external feedback mechanisms such as chatbot interventions. These findings suggest that tailored interventions may play a crucial role in bridging information gaps and promoting sustainable behavior change in populations with limited health education access.

This study did not aim to compare the effectiveness of specific intervention type across different behavioral trajectories but rather to explore how tailored intervention influenced behavioral adaptation. Given the total sample dispersed across five feedback conditions, this study was under-powered to detect small-to-moderate main effects of individual intervention types, raising the possibility of a Type II error. Moreover, the single-arm, repeated-measures design—without an untreated control arm—prevents full exclusion of natural temporal trends or external influences and limits causal inference. Additionally, the intervention algorithm did not fully account for individual characteristics (e.g., motivation, prior dietary habits, and psychological factors) [36], nor did it capture family- (e.g., parental modelling) [37] and community-level influences (e.g., access to convenience stores and drink shops in the community) [38], both of which are established determinants of adolescent SSB intake. Future research should incorporate an untreated concurrent control or an appropriately matched historical control. It should also investigate how intervention strategies interact with trajectory-based consumption patterns, individual characteristics, and modifiable contextual factors to optimize the personalization of digital health interventions.

## Conclusions

This study utilized trajectory analysis to investigate how adolescents’ sugar intake changed over time in response to a tailored sugar reduction intervention. By identifying three distinct intake trajectories, the findings revealed that intervention effects were not uniform but dynamic, reinforcing the evidence that behavioral change is not an immediate outcome of a single intervention but rather a gradual process shaped through repeated interactions. These findings emphasize the need for adaptive feedback strategies, particularly considering variations in response speed and behavioral adaptation patterns. Although the study did not directly compare intervention effects across trajectory groups, individuals with higher baseline intake showed a more pronounced response to personalized intervention. Furthermore, reinforcement-based tailored feedback appeared to prevent recurrences of increased intake and helped stabilize consumption levels.

Given that SSB consumption remains a major public health concern, future intervention strategies should prioritize sustainable behavioral change through gradual, adaptive approaches tailored to individual responses. In addition, the observed differences in intervention receptivity between Korean native and racial and ethnic adolescents underscore the need to consider individual characteristics to enhance accessibility and effectiveness across diverse populations.

## Supporting information

S1 Table GBTM model comparison (2–4 classes): BIC and APPA per group. Note. BIC: Bayesian Information Criterion; APPA: Average Posterior Probability of Assignment. a Although the 2‑class solution showed the best fit by BIC, it essentially split participants into ‘consumers vs non‑consumers,’ failing to capture behavioral heterogeneity; therefore, we selected the 3‑class model to balance fit and interpretability. Simulation-based class-recovery (1,000 simulations): three-class structure recovered 87.2%.
(DOCX)

S2 Table Estimated effect of the intervention based on linear mixed effect model in racial and ethnic adolescents, including parental foreign status (n = 21). Note. Reference groups: Feedback type (advanced education), Sugar intake trajectories (maintenance), Gender (boy), Socioeconomic status (≥moderate), Academic status (≥moderate), Parental foreign status (One foreign parent). Intervention refers to the application of tailored-feedback via the R-Ma Bot during the 14-day period. Group refers to the sugar intake trajectory group (maintenance, reduction, or no-intake).
(DOCX)

## Abbreviations

SSB: sugar-sweetened beverage
GBTM: group-based trajectory modeling
R-Ma Bot: read and manage your health robot
