# Supplementary material for: Sugar intake trajectories in adolescents: Evaluating behavioral change with group-based trajectory modeling
Source: PLoS One. 2025 Sep 30;20(9):e0333389. doi: 10.1371/journal.pone.0333389 (PMC12483263; doi:10.1371/journal.pone.0333389)
Supplement: S1 Table — Note. BIC: Bayesian Information Criterion; APPA: Average Posterior Probability of Assignment. a Although the 2‑class solution showed the best fit by BIC, it essentially split participants into ‘consumers vs non‑consumers,’ failing to capture behavioral heterogeneity; therefore, we selected the 3‑class model to balance fit and interpretability. Simulation-based class-recovery (1,000 simulations): three-class structure recovered 87.2%. (DOCX) [file pone.0333389.s001.docx]

**S1 Table. GBTM model comparison (2-4 classes): BIC and APPA per group**

| **Model** | **Number of Groups** | **BIC** | **APPA per Group** |
| --- | --- | --- | --- |
| 2-group | 2 | –1321.505 | (1.000, 1.000) |
| 3-group | 3 | –1305.171 | (0.859, 0.730, 1.000) ^a^ |
| 4-group | 4 | –1280.713 | (0.826, 0.780, 0.936, 1.000) |

Note: BIC: Bayesian Information Criterion; APPA: Average Posterior Probability of Assignment.

^a^ Although the 2‑class solution showed the best fit by BIC, it essentially split participants into ‘consumers vs non‑consumers,’ failing to capture behavioral heterogeneity; therefore, we selected the 3‑class model to balance fit and interpretability. Simulation-based class-recovery (1,000 simulations): three-class structure recovered 87.2%.
