# Supplementary material for: Sugar intake trajectories in adolescents: Evaluating behavioral change with group-based trajectory modeling
Source: PLoS One. 2025 Sep 30;20(9):e0333389. doi: 10.1371/journal.pone.0333389 (PMC12483263; doi:10.1371/journal.pone.0333389)
Supplement: S2 Table — Note. Reference groups: Feedback type (advanced education), Sugar intake trajectories (maintenance), Gender (boy), Socioeconomic status (≥moderate), Academic status (≥moderate), Parental foreign status (One foreign parent). Intervention refers to the application of tailored-feedback via the R-Ma Bot during the 14-day period. Group refers to the sugar intake trajectory group (maintenance, reduction, or no-intake). (DOCX) [file pone.0333389.s002.docx]

**S2 Table. Estimated effect of the intervention based on linear mixed effect model in racial and ethnic adolescents, including parental foreign status (n=21)**

| Variables | Model 1 | | | Model 2 | | | Model 3 | | |
| --- | --- | --- | --- | --- | --- | --- | --- | --- | --- |
|  | β | SE | *p* | β | SE | *p* | β | SE | *p* |
| Intercept | 258.51 | 136.83 | .083 | 256.54 | 134.87 | .081 | 252.84 | 141.36 | .099 |
| Feedback type |  |  |  |  |  |  |  |  |  |
| Strong reward | 44.97 | 39.86 | .261 | 33.21 | 48.56 | .495 | 31.18 | 47.84 | .515 |
| Moderate reward | 47.92 | 40.24 | .235 | 39.24 | 49.00 | .424 | 31.22 | 48.32 | .519 |
| Minimal reward | -1.41 | 44.88 | .975 | -11.25 | 52.97 | .832 | -4.32 | 52.00 | .934 |
| Basic education | 23.50 | 39.63 | .554 | 13.54 | 48.42 | .780 | 10.35 | 47.55 | .828 |
| Sugar intake trajectories |  |  |  |  |  |  |  |  |  |
| Reduction | 6.39 | 9.99 | .523 | 10.77 | 11.53 | .351 | 32.24 | 19.79 | .105 |
| No-Intake | -8.74 | 23.72 | .713 | -10.14 | 23.46 | .666 | -11.22 | 32.16 | .728 |
| Gender |  |  |  |  |  |  |  |  |  |
| Girl | 6.43 | 11.40 | .574 | 5.86 | 11.26 | .604 | 8.03 | 11.81 | .498 |
| Age (year) | -16.74 | 8.30 | .045 | -16.67 | 8.18 | .043 | -17.16 | 8.60 | .047 |
| Socioeconomic status |  |  |  |  |  |  |  |  |  |
| Low | -9.20 | 10.84 | .397 | -9.81 | 10.70 | .361 | -8.61 | 11.21 | .444 |
| Academic status |  |  |  |  |  |  |  |  |  |
| Low | -14.43 | 13.66 | .292 | -13.35 | 13.53 | .325 | -17.62 | 14.21 | .216 |
| Body mass index (kg/m^2^) | 2.00 | 1.86 | .284 | 1.94 | 1.84 | .291 | 2.10 | 1.93 | .277 |
| Parental foreign status |  |  |  |  |  |  |  |  |  |
| Both foreign parent | 8.50 | 13.98 | .544 | 8.61 | 13.78 | .533 | 7.72 | 14.46 | .594 |
| Intervention*time |  |  | .004 |  |  | .006 |  |  | .005 |
| Intervention*group |  |  |  |  |  | .865 |  |  | .657 |
| Intervention*time*group | |  |  |  |  |  |  |  | .014 |

Note. Reference groups: Feedback type (advanced education), Sugar intake trajectories (maintenance), Gender (boy), Socioeconomic status (≥moderate), Academic status (≥moderate), Parental foreign status (One foreign parent). *Intervention* refers to the application of tailored-feedback via the R-Ma Bot during the 14-day period. *Group* refers to the sugar intake trajectory group (maintenance, reduction, or no-intake).
